# Supplementary material for: Item response theory model highlighting rating scale of a rubric and rater–rubric interaction in objective structured clinical examination
Source: PLoS One. 2024 Sep 6;19(9):e0309887. doi: 10.1371/journal.pone.0309887 (PMC11379165; doi:10.1371/journal.pone.0309887)
Supplement: Attachment — Submitted filename: rebuttal_letter.pdf [file pone.0309887.s001.pdf]

Response to Reviewers

Editor:

Your paper was reviewed by two reviewers. They suggested some additional comments to improve paper quality. Overall, there are just minor issues which could be revised.

Authors response:

Thank you for your consideration of our manuscript. We have revised the manuscript in accordance with the comments raised by the two reviewers. Our point-by-point responses to these comments are presented below, and newly added or revised text is indicated by blue font in the revised manuscript with tracked changes.

Reviewer #1:

This is an excellent manuscript that advances the work on reliability and validity of rater evaluation methods. It is a creative application of a standardized methodology to an important clinical process, one that is broadly utilized throughout the world. The methods are sound, the results and analysis are clear, and the authors have done an excellent job considering and accounting for any potential pitfalls and confounds. It would be interesting to augment the manuscript to clarify how this method might or might not improve clinical practice down the line, whether there is any implication for other branches of clinimetrics, and whether this approach is applicable to evaluations of patient treatment in routine care. Overall, this is an interesting analysis and an important application of rigorous analytic techniques to improve medical education and clinical training. I look forward to its publication and to potential dissemination across different domains.

Authors response:

Thank you for your careful review and valuable suggestion. Although the direct contribution of our model to general clinical practice, other branches of clinimetrics, and routine patient care evaluations may be limited, our model has the following features, which are relevant to the field of clinimetrics and will ultimately lead to higher quality clinical practice and patient care:

- **Appropriate Selection of Competent Professionals:** Our model realizes accurate measurement of examinee abilities in the OSCE, even when multiple raters are

involved. This contributes to the appropriate selection of competent medical and dental professionals, enhancing future patient care quality.

- **Trust in Evaluation and Learning Processes:** Accurate ability measurement enhances the trustworthiness of both the evaluation and the learning processes for medical and dental students. This trust is fundamental for the credibility and effectiveness of medical education.
- **Improved Rubric Design and Development:** Our model allows for a detailed analysis of the characteristics of each evaluation item in the rubric. This helps in refining and developing more effective rubrics, thereby ensuring fair and comprehensive assessment criteria.
- **Enhanced Rater Training:** Our model provides detailed and objective information on the characteristics of raters. Offering such information to each rater serves as valuable feedback for rater training programs. Such feedback can help raters, who are typically medical and dental professionals, become aware of their biases and standardize their evaluations, thereby improving the reliability of assessments.
- **Reducing Rating Cost:** By enabling high-quality rubric design and improving rater reliability, our model can potentially reduce the number of raters and stations while maintaining ability measurement accuracy, which contributes to cost reduction for managing examinations.
- **Broader Applicability:** Our model is applicable to various rubric-based performance assessments beyond OSCEs, including writing exams, interview exams, and presentation exams, in fields such as psychology, nursing, and allied health professions.

We have added these points to the “Implications of Findings” section in the revised manuscript. Please see the blue text in the section.

Reviewer #2:

Authors response:

Thank you very much for your careful review and valuable suggestions. We have revised our manuscript based on your comments. Please find our point-by-point responses below. Newly added and revised text are indicated by blue font in the revised manuscript.

Comment 1:

Please discuss the applicability of the models discussed.

Authors response:

The proposed IRT model is applicable to various rubric-based performance assessments, including OSCEs, and offers numerous benefits as detailed in our response to reviewer 1. However, to use our model appropriately, careful rater allocation is required for IRT parameter linking.

The ideal condition for parameter linking is a setting where all examinees are assessed by all raters. However, in general settings, to reduce the scoring burden on each rater, each examinee is assessed by a few different raters from a pool of raters. To ensure parameter linking in such cases, each examinee must be assessed by at least two raters, and the combinations of raters must be changed several times throughout the examination. This is because when raters remain fixed, the factors of raters and examinees become nested, making it impossible to separate their characteristics. Under inappropriate rater allocation designs, fair ability measurement cannot be achieved by any statistical analysis method, including IRT, without some strong assumptions. For appropriate rater allocation designs, please refer to Eckes (2015). Note that our dataset follows an appropriate rater allocation design.

We have clarified these points in the "Limitations and Future Works" section.

Comment 2:

Please Simplify your results if possible in order to be understandable for all readers.

Authors response:

Thank you for the suggestion. We have carefully reconsidered the contents of the results section and concluded that all the experiments described are crucial for validating the effectiveness of our proposed model. Although most of the descriptions are necessary to ensure reliability and reproducibility, we have removed some theoretical details in the section labeled "Model comparison using information criteria" (please see the blue text). These modifications have helped to shorten the text slightly while maintaining the accuracy of the descriptions. We believe that further simplification would hinder the ability of readers who are not familiar with statistical methodologies to correctly interpret the meaning of the presented data and results.

Comment 3:

Simplify your conclusion to make it clear about the comparison of the rating models and the assumed new models.

Authors response:

In accordance with your comment, we have simplified the conclusion by focusing on how the proposed model improves upon conventional models. Please see the blue text in the revised manuscript for details.

Journal Requirements:

When submitting your revision, we need you to address these additional requirements.

Comment 1:

Please ensure that your manuscript meets PLOS ONE's style requirements, including those for file naming. The PLOS ONE style templates can be found at https://journals.plos.org/plosone/s/file?id=wjVg/PLOSOne_formatting_sample_main_body.pdf and https://journals.plos.org/plosone/s/file?id=ba62/PLOSOne_formatting_sample_title_authors_affiliations.pdf.

Authors response:

We have reviewed the style guidelines and confirmed that the paper follows the template.

Comment 2:

Please note that PLOS ONE has specific guidelines on code sharing for submissions in which author-generated code underpins the findings in the manuscript. In these cases, all author-generated code must be made available without restrictions upon publication of the work. Please review our guidelines at https://journals.plos.org/plosone/s/materials-and-software-sharing#loc-sharing-code and ensure that your code is shared in a way that follows best practice and facilitates reproducibility and reuse.

Authors response:

Thank you for the instructions. We have switched to a recommended repository for sharing our code and data. Please see our response to comment 6 below for further details.

Comment 3:

Please provide additional details regarding participant consent. In the ethics statement in the Methods and online submission information, please ensure that you have specified (1) whether consent was informed and (2) what type you obtained (for instance, written or verbal, and if verbal, how it was documented and witnessed). If your study included minors, state whether you obtained consent from parents or guardians. If the need for consent was waived by the ethics committee, please include this information. If you are reporting a retrospective study of medical records or archived samples, please ensure that you have discussed whether all data were fully anonymized before you accessed them

and/or whether the IRB or ethics committee waived the requirement for informed consent. If patients provided informed written consent to have data from their medical records used in research, please include this information.

Authors response:

Thank you for the instructions. The need for consent was waived by the ethics committee. Please see our response to comment 7 below for further details.

Comment 4:

Thank you for stating the following financial disclosure: [This work was supported by JSPS KAKENHI Grant Number 19H05663.]. Please state what role the funders took in the study. If the funders had no role, please state:

"The funders had no role in study design, data collection and analysis, decision to publish, or preparation of the manuscript."

If this statement is not correct you must amend it as needed. Please include this amended Role of Funder statement in your cover letter; we will change the online submission form on your behalf.

Authors response:

As you correctly noted, the funder had no role in study design, data collection and analysis, decision to publish, or preparation of the manuscript. We have added this information to the cover letter accordingly.

Comment 5:

Please expand the acronym "JSPS" (as indicated in your financial disclosure) so that it states the name of your funders in full. This information should be included in your cover letter; we will change the online submission form on your behalf.

Authors response:

The acronym JSPS stands for "Japan Society for the Promotion of Science". We have added the full name to the cover letter accordingly.

Comment 6:

Thank you for uploading your study's underlying data set. Unfortunately, the repository you have noted in your Data Availability statement does not qualify as an acceptable data repository according to PLOS's standards. At this time, please upload the minimal data

set necessary to replicate your study's findings to a stable, public repository (such as figshare or Dryad) and provide us with the relevant URLs, DOIs, or accession numbers that may be used to access these data. For a list of recommended repositories and additional information on PLOS standards for data deposition, please see https://journals.plos.org/plosone/s/recommended-repositories.

Authors response:

In response to your comments 2 and 6, we have decided to use Dryad from the list of recommended repositories. We have uploaded the minimal dataset and software to the repository at the following DOI:
https://doi.org/10.5061/dryad.tmpg4f56q

Comment 7:

Please include your full ethics statement in the 'Methods' section of your manuscript file. In your statement, please include the full name of the IRB or ethics committee who approved or waived your study, as well as whether or not you obtained informed written or verbal consent. If consent was waived for your study, please include this information in your statement as well.

Authors response:

Our study was waived by the ethics committee. Therefore, we have included the following statement, along with the full name of the ethics committee (the Dental Research Ethics Committee of Tokyo Medical and Dental University), in the ethics statement of the online submission form:

> *The Dental Research Ethics Committee of Tokyo Medical and Dental University has stated in writing that this research falls outside the purview of the Ethical Guidelines for Medical Research Involving Human Subjects and is thus deemed 'not applicable.' This decision is documented in the minutes of the 2019 9th Ethics Review Committee meeting. The videos recorded at the medical interview tests were used secondarily for this study. The students were informed in writing of their right to refuse secondary use of the recorded videos for this study.*

We have also added the following statement in the Method and Materials section:

> *The videos recorded at the medical interview tests for educational purpose were used secondarily for this study. The students were informed in writing of their right to refuse secondary use of the recorded videos for this study. The Dental Research Ethics Committee of Tokyo Medical and Dental University has stated in writing that this*

research falls outside the purview of the Ethical Guidelines for Medical Research Involving Human Subjects and is thus deemed 'not applicable.' This decision is documented in the minutes of the 2019 9th Ethics Review Committee meeting.

Comment 8:

Please review your reference list to ensure that it is complete and correct. If you have cited papers that have been retracted, please include the rationale for doing so in the manuscript text, or remove these references and replace them with relevant current references. Any changes to the reference list should be mentioned in the rebuttal letter that accompanies your revised manuscript. If you need to cite a retracted article, indicate the article's retracted status in the References list and also include a citation and full reference for the retraction notice.

Authors response:

We have reviewed the reference list and confirmed that all the cited papers are appropriately listed. We have modified the reference list as follows:

- For citations 2, 4, 9, 12, 13, 24, 46, 48, and 55, we have added missing information, including issue numbers and pages, and made style changes in accordance with the journal's guidelines.
- The following two citations have been removed in response to comment 2 from reviewer 2:
  - Akaike H. A new look at the statistical model identification. IEEE Trans Automat Contr. 1974;19(6):716–723.
  - Schwarz G. Estimating the dimensions of a model. Ann Stat. 1978;6:461–464.
